# Spatial proximity effects on cognitive processing of multimedia learning among college students: evidence from functional near-infrared spectroscopy

**DOI:** 10.3389/fpsyg.2025.1559696

**Published:** 2025-08-06

**Authors:** Yan Ma, Qiuyue Peng, Bowen Long

**Affiliations:** ^1^Wisdom Education Research Institute, Chongqing Normal University, Chongqing, China; ^2^College of Computer and Information Science, Chongqing Normal University, Chongqing, China

**Keywords:** spatial proximity effect, cognitive processing, fNIRS, multimedia learning, online learning, distraction effect

## Abstract

**Aim:**

From a cognitive perspective, the spatial proximity effect suggests that during the process of the brain processing information, the degree of information processing and construction varies with spatial distance. However, most previous studies mainly relied on behavioral experiments.

**Methods:**

To investigate the neural mechanisms underlying the spatial proximity effect, this study employed functional near-infrared spectroscopic imaging (fNIRS) to monitor and analyze the neural activity in the prefrontal cortex of 36 college students while they engaged in learning tasks with different graphic formats (proximity vs. separation). In addition, the study also explored how disciplinary differences affect the strength of spatial proximity effects and corresponding brain activation patterns.

**Results:**

The findings revealed that activation levels in the middle temporal gyrus, frontal pole, and dorsolateral prefrontal cortex were significantly higher in the spatial proximity condition compared to the spatial separation condition, accompanied by an improved rate of correct answers. These results suggest that the spatial proximity of illustrations and text can significantly enhance learning outcomes. Furthermore, when the subjects were learning physics, the activation level of the superior temporal gyrus was significantly higher than that when they were learning English and geography. Moreover, under the adjacent condition, the accuracy rate of learning performance was higher, which reveals that the spatial proximity effect is significant under the conditions of learning English, Geography, and Physics and that the superior temporal gyrus may be related to the neural mechanism of the spatial proximity effect in different disciplines.

**Conclusion:**

This study provides evidence for the neural mechanisms behind the spatial proximity effect and verifies the influence of different disciplines on this effect. Provided a practical basis for media teaching design from the perspective of cognitive processing.

## 1 Introduction

Multimedia learning involves the cognitive processing and construction of verbal and pictorial representations (Yang et al., 2023). Simultaneous presentation of textual and pictorial information in instructional settings enhances learning outcomes (Mayer, 2002). Mayer defines multimedia as the combined presentation of text and images, in which text encompasses printed or spoken forms and images refer to visual materials (Lawson et al., 2021). According to Mayer's Cognitive Theory of Multimedia Learning (CTML), multimedia instructional information involves the combined presentation of text and images to facilitate learning. It emphasizes the learner's process of “selecting, organizing, and integrating graphic and textual information” (Mayer, 2014). However, multimedia effectiveness depends on content characteristics, representational formats, and the spatial arrangement of elements (Vu et al., 2021). Consequently, multimedia messages should align with working memory structure to enable efficient cognitive processing (Mutlu-Bayraktar et al., 2019). The spatial proximity effect refers to the phenomenon in which the spatial closeness of graphic and textual elements facilitates learners' knowledge construction and enhances learning effectiveness (Mayer, 2005a,b). Schroeder and Cenkci (2018) through meta-analysis, confirmed the significant impact of this effect in multimedia learning. They attributed its efficacy to the facilitation of information processing and integration when elements are spatially proximal (Clark and Mayer, 2023; Castro-Alonso et al., 2021). Beege et al. (2019) reported that a moderate distance (4.2–4.3 cm) between graphic and textual elements yields the most favorable learning outcomes.

The spatial proximity effect has been validated across various disciplines, including Geography (Makransky et al., 2019), Physics (de Koning et al., 2020), and Mechanical Engineering (Mayer and Moreno, 1998); however, most studies have relied on subjective measurement methods (Wang et al., 2016). Tarmizi and Sweller (1988) found that the integrated presentation of information during mathematical problem-solving reduces cognitive load, thereby enhancing learning (Tarmizi and Sweller, 1988). Meta-analyses conducted by Schroeder and Cenkci (2018) demonstrated that multimedia integration design not only enhances learning efficiency but also optimizes knowledge organization through mental model construction, thereby promoting deep learning. Consequently, the spatial proximity effect is a critical factor that must be considered in multimedia learning and instructional design. Proximity refers to the closeness of two events in the physical dimensions of time and space (Herrera et al., 2022). In this study, proximity is manipulated by adjusting the spatial layout of graphics and text. In the spatial proximity condition, the text is positioned adjacent to the corresponding area of the image, whereas in the spatial separation condition, the image is placed on the left side of the page, and the text is positioned on the right.

Theoretical explanations for the spatial proximity effect vary across different perspectives. Schroeder and Cenkci (2020) argue, from the cognitive load perspective, that the parallel presentation of multiple information sources may distract attention and increase cognitive load, as it requires additional effort to search for and integrate relevant content from separated text and images. The cognitive theory of multimedia learning, proposed by Mayer, is a learning framework based on human information processing mechanisms. It explains how learners integrate knowledge through text and images and emphasizes that instructional design should align with cognitive principles to promote meaningful learning (Mayer, 2005a,b, 2024). The core concepts of this theory are grounded in the dual-channel assumption, the capacity-limited assumption, and the active processing assumption. It proposes several design principles, including the multimedia principle, the spatial and temporal proximity principle, the channelization principle, the redundancy principle, and the coherence principle. This study specifically focuses on the spatial proximity principle as applied to graphic materials (Mayer and Moreno, 1998; Mayer, 2011; Mayer and Pilegard, 2005). According to this theory, the spatial proximity of graphic and textual materials can more effectively promote learners‘ integrated knowledge processing. When corresponding text and images are presented in close spatial proximity, synchronized working memory processing can be achieved, which reduces distraction and facilitates the construction of an integrated mental model. Therefore, the design of multimedia materials should fully consider material characteristics and cognitive principles to effectively reduce learners' cognitive load (Juanjuan et al., 2021).

Traditional behavioral experiments assess learners' cognitive states through external observation, primarily measuring final learning outcomes (e.g., test scores) and focusing solely on the cognitive domain level (Kessels, 2019). Learners' internal cognitive processes involved in processing graphic information cannot be directly observed, making it difficult to precisely distinguish the mechanisms underlying mental representation integration and to deeply explore the cognitive processing mechanisms occurring during learning. For example, spatial proximity of graphics can improve test scores; however, behavioral data alone cannot determine whether this improvement results from increased visual search efficiency or reduced working memory load (Frick and Schüler, 2023). Therefore, it is essential to assess the cognitive integration processes in multimedia learning using physiological indicators, which can overcome the limitations of behavioral experiments and enable the observation of learners' implicit cognitive and neural mechanisms. In recent years, physiological measurement techniques such as eye tracking and EEG have been increasingly employed in multimedia learning research due to their effectiveness in capturing cognitive information. For instance, Altan and Cagiltay (2015) observed that although no significant differences in learning performance, gaze frequency, or gaze duration were found between groups, the separated group invested more time and cognitive effort during learning. Similarly, Makransky et al. (2019) found that the spatial proximity group achieved higher test scores and experienced lower cognitive load, despite no significant differences in alpha-band brainwave activity. However, although existing behavioral studies (e.g., time investment, cognitive load) have demonstrated that spatial proximity influences cognitive load and learning performance, there remains a lack of neural evidence supporting a causal relationship between neural activity and learning outcomes. Additionally, there is insufficient evidence regarding differences in activation patterns of specific brain regions under varying spatial conditions, and it is unclear whether spatial proximity or separation significantly alters the intensity or patterns of neural activation.

With the advancement of learning sciences and intelligent technologies, brain sciences have emerged as a critical perspective for understanding learners' motivation and cognition (Luria et al., 2021), shifting research paradigms from behaviorism and cognitivism to neuroscience (Zhang et al., 2024). Common brain imaging techniques used in educational research include electroencephalography (EEG), functional near-infrared spectroscopy (fNIRS), and functional magnetic resonance imaging (fMRI). Functional near-infrared spectroscopy (fNIRS), with its non-invasive, high-resolution, and cost-effective characteristics (Pinti et al., 2020), has been proven suitable for research in authentic educational contexts (Babiloni and Astolfi, 2014). Compared to EEG, fNIRS is more effective in detecting the spatial characteristics of neural activity during cognitive processing. Functional magnetic resonance imaging (fMRI) presents limitations such as high cost and immobility, and its sensitivity to motion artifacts frequently restricts its application in dynamic environments (Yang and Wang, 2025). The noiseless and non-enclosed nature of fNIRS enables subjects to complete data acquisition in a near-natural reading state, providing higher ecological validity and greater practical feasibility. Therefore, fNIRS overcomes the major limitations of EEG and fMRI in dynamic learning tasks and is particularly suitable for investigating the neural mechanisms underlying the spatial proximity effect in multimedia learning. Studies have shown that higher working memory load tends to elicit greater activation within the prefrontal cortex (Csipo et al., 2021); and cognitive processing in working memory (e.g., information retention, manipulation, and integrative processing) significantly increases activation in the dorsolateral prefrontal cortex and the frontal pole (Kim et al., 2015). As a result, learners are more likely to form mental connections when images are presented in close proximity, whereas when images are separated, they tend to expend additional cognitive resources to search for the corresponding information (Mayer, 2005a,b). Chen et al. (2022) used fNIRS to investigate the spatial proximity effect in ancient poetry multimedia learning. They found that even when the materials contained redundancy, the spatial proximity effect remained significant, with greater activation observed in the left frontal lobe and bilateral dorsolateral prefrontal cortex. These brain regions may constitute the neural basis of this effect of their findings. However, the specific focus on ancient poetry limits the generalizability of these findings.

This study aims to employ fNIRS to investigate learners' cognitive processing under the multimedia spatial proximity effect and to examine the influence of disciplinary differences on the magnitude of this effect. By integrating behavioral and neuroimaging data, this study aims to elucidate the mechanisms underlying the spatial proximity effect and to provide an objective and scientific basis for multimedia instructional design. Based on the literature reviewed above, the following hypotheses are proposed:

H1: Subjects in the spatial proximity condition are expected to exhibit higher test accuracy, retention, and transfer scores, as well as shorter response times. This effect is attributed to the reduction of cognitive load associated with visual search and information integration facilitated by spatial proximity.
H2: The spatial proximity condition is hypothesized to significantly reduce activation levels in brain regions associated with executive function and working memory [e.g., dorsolateral prefrontal cortex (dlPFC), frontal pole cortex (FPC)], indicating that proximity presentation reduces the additional cognitive effort required to integrate dispersed information.
H3: Activation of brain regions involved in automated semantic integration [e.g., middle temporal gyrus (MTG)] is expected to be significantly enhanced in the spatial proximity condition, indicating that spatial proximity facilitates more fluent processing of graphic information.
H4: Activation of brain regions associated with semantic integration and spatial concept mapping [e.g., superior temporal gyrus (STG), Broca's area] is expected to be significantly enhanced in the spatial separation condition among learners of highly interactive disciplines (e.g., physics).

## 2 Method

### 2.1 Participants

The sample size was estimated using G^*^Power 3.1.9 software, based on effect sizes reported in previous fNIRS studies (Chen et al., 2022; Zhang et al., 2025; Lei et al., 2021). Using a significance level of α = 0.05, a medium effect size of *d* = 0.25, and a statistical power of 0.80, the calculation indicated that a minimum of 24 participants was required. Considering the substantial inter-individual variability in fNIRS data (e.g., poor data quality), the study recruited 36 college students from a university in Chongqing, comprising 19 females and 17 males, with an age range of 22 to 28 years (M ± SD, 22.3 ± 1.69). All participants were native Chinese speakers and used English as a second language. To control for language proficiency and prior English knowledge, participants were required to meet one of the following criteria: (a) pass the CET-4 examination; (b) self-assess their English proficiency as “good” or higher and pass an English comprehension screening, which involved reading a passage of equivalent difficulty to the experimental materials with a minimum comprehension accuracy of 80%. To control for prior knowledge in the subject areas (physics/geography), participants were required to complete a pre-test questionnaire before the experiment, including items such as “I have studied the working principles of electromagnetic relays” and “I understand the formation mechanisms of thermal circulation.” The questionnaire employed a Likert scale (1 = completely unaware, 5 = completely proficient), and eligible participants scored no higher than 2 on each item. All participants were screened using the Edinburgh Handedness Questionnaire to ensure that they were strongly right-handed, had normal or corrected-to-normal vision, were free from color blindness, color weakness, and other visual impairments, and had no history of mental illness. The 36 participants were randomly assigned to either the spatial proximity group (control group 1) or the spatial separation group (control group 2), with 18 participants in each condition. Informed written consent was obtained from all participants, and they were explicitly informed that they could withdraw from the experiment at any time without penalty.

### 2.2 Arrangement of experimental apparatus and probes

This experiment employed a near-infrared spectral brain imaging system (NIRSmart, Danyang Huichuang), which utilizes near-infrared light at two distinct wavelengths: 730 nm and 850 nm. Spectral signals were recorded at a sampling rate of 11 Hz. Changes in oxygenated hemoglobin (HbO) and deoxygenated hemoglobin (HbR) concentrations were calculated using the modified Beer-Lambert law. The instrument operates on three-dimensional positioning principles and employs the international 10/20 electrode placement system for spatial localization. The coordinates of each channel and the corresponding brain region calibration information are presented in Table 1.

**Table 1:** Correspondence between spatial registration information of 48 NIRS channels in the experiment and Brodmann areas.

**Channel**	**MNI coordinates**			**Region (Brodmann areas)**	**Region (lobar division)**	**Proportion**
	**x**	**Y**	**z**			
CH1(S1-D1)	69	–12	–6	21 - Middle temporal gyrus	Temporal lobe	0.98
CH2 (S1-D6)	71	–27	12	22 - Superior temporal gyrus	Temporal lobe	0.44
CH3 (S2-D1)	59	17	1	38 - Temporopolar area	Temporal lobe	0.54
CH4 (S2-D2)	54	44	1	47 - Inferior prefrontal gyrus	Temporal lobe	1
CH5 (S2-D7)	59	29	16	47 - Inferior prefrontal gyrus	Prefrontal Cortex	0.62
CH6 (S3-D2)	38	63	5	10 - Frontopolar area	Prefrontal Cortex	0.60
CH7 (S3-D3)	14	73	8	11 - Orbitofrontal area	Frontal lobe(Orbital)	0.52
CH8 (S3-D8)	26	64	24	10 - Frontopolar area	Prefrontal Cortex	1
CH9 (S4-D3)	−15	73	7	11 - Orbitofrontal area	Frontal lobe(Orbital)	0.62
CH10 (S4-D4)	−41	62	1	11 - Orbitofrontal area	Frontal lobe(Orbital)	0.49
CH11 (S4-D9)	−28	63	22	10 - Frontopolar area	Prefrontal Cortex	0.98
CH12 (S5-D4)	–53	46	–1	38 - Temporopolar area	Temporal lobe	0.59
CH13 (S5-D5)	–63	7	–9	21 - Middle temporal gyrus	Temporal lobe	0.99
CH14 (S5-D10)	–61	24	12	22 - Superior temporal gyrus	Temporal lobe	0.43
CH15 (S6-D5)	–71	–18	–9	21 - Middle temporal gyrus	Temporal lobe	0.93
CH16 (S6-D11)	–72	–34	8	21 - Middle temporal gyrus	Temporal lobe	0.75
CH17 (S7-D1)	67	1	16	22 - Superior temporal gyrus	Temporal lobe	0.63
CH18 (S7-D6)	68	–14	31	43 - Subcentral area	Frontal lobe (Opercular)	0.37
CH19 (S7-D7)	63	10	31	44 - Pars opercularis_ part of Broca's area	Frontal lobe (Broca's area)	0.57
CH20 (S7-D12)	63	–10	43	6 - Pre-motor and supplementary motor cortex	Frontal lobe (Motor)	0.74
CH21 (S8-D2)	46	50	18	10 - Frontopolar area	Prefrontal cortex	0.47
CH22 (S8-D7)	51	35	32	46 - Dorsolateral prefrontal cortex	Prefrontal cortex	0.77
CH23 (S8-D8)	34	49	35	10 - Frontopolar area	Prefrontal cortex	0.93
CH24 (S8-D13)	41	33	46	46 - Dorsolateral prefrontal cortex	Prefrontal cortex	0.77
CH25 (S9-D3)	–1	65	24	10 - Frontopolar area	Prefrontal cortex	0.87
CH26 (S9-D8)	13	59	40	10 - Frontopolar area	Prefrontal cortex	1
CH27 (S9-D9)	–14	59	39	10 - Frontopolar area	Prefrontal cortex	1
CH28 (S9-D14)	–2	47	51	10 - Frontopolar area	Prefrontal cortex	0.99
CH29 (S10-D4)	–49	50	19	47 - Inferior prefrontal gyrus	Prefrontal cortex	0.59
CH30 (S10-D9)	–38	48	33	10 - Frontopolar area	Prefrontal cortex	0.91
CH31 (S10-D10)	–55	30	27	45 - pars triangularis Broca's area	Frontal lobe (Broca's area)	0.65
CH32 (S10-D15)	–45	32	43	46 - Dorsolateral prefrontal cortex	Prefrontal cortex	0.87
CH33 (S11-D5)	–68	–5	10	21 - Middle temporal gyrus	Temporal lobe	0.59
CH34 (S11-D10)	–65	6	26	22 - Superior temporal gyrus	Temporal lobe	0.51
CH35 (S11-D11)	–69	–20	27	42 - Primary and auditory association cortex	Temporal lobe	0.67
CH36 (S11-D16)	–66	–15	39	43 - Subcentral area	Frontal lobe (Opercular)	0.46
CH37 (S12-D7)	53	17	44	45 - pars triangularis Broca's area	Prefrontal cortex	0.36
CH38 (S12-D12)	53	–6	55	9 - Dorsolateral prefrontal cortex	Prefrontal cortex	0.51
CH39 (S12-D13)	43	17	57	9 - Dorsolateral prefrontal cortex	Prefrontal cortex	0.64
CH40 (S13-D8)	21	44	50	10 - Frontopolar area	Prefrontal cortex	0.89
CH41 (S13-D13)	27	28	59	9 - Dorsolateral prefrontal cortex	Prefrontal cortex	0.89
CH42 (S13-D14)	12	34	61	9 - Dorsolateral prefrontal cortex	Prefrontal cortex	0.83
CH43 (S14-D9)	–25	42	49	10 - Frontopolar area	Prefrontal cortex	0.92
CH44 (S14-D14)	–14	33	61	9 - Dorsolateral prefrontal cortex	Prefrontal cortex	0.84
CH45 (S14-D15)	–33	26	57	9 - Dorsolateral prefrontal cortex	Prefrontal cortex	0.85
CH46 (S15-D10)	–56	13	39	45 - pars triangularis Broca's area	Frontal lobe (Broca's area)	0.41
CH47 (S15-D15)	–47	15	54	9 - Dorsolateral prefrontal cortex	Prefrontal cortex	0.61
CH48 (S15-D16)	–57	–10	51	6 - Pre-motor and supplementary motor cortex	Frontal lobe (Motor)	0.81

**Figure 1:**
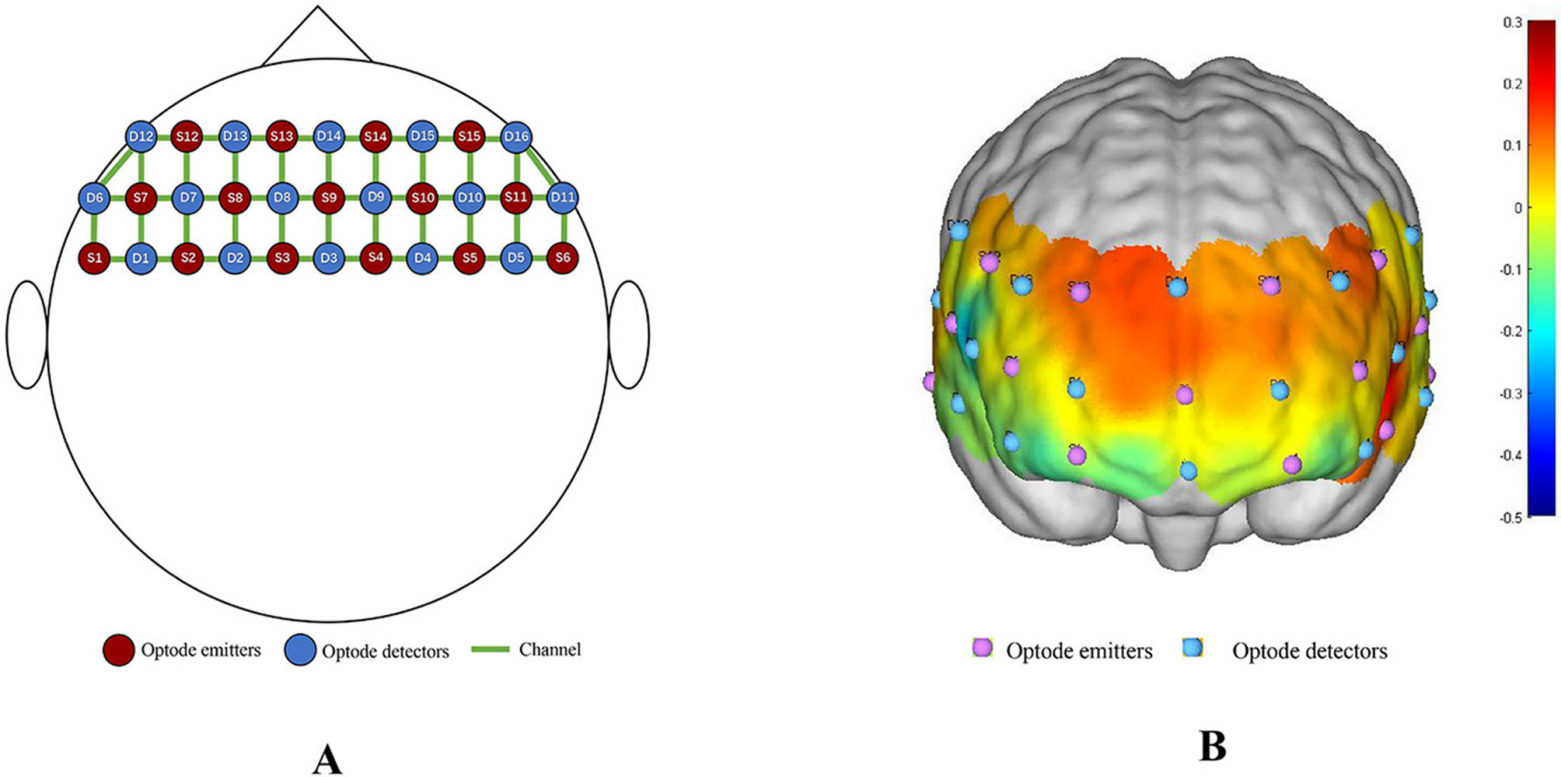
fNIRS channel layout diagram with optode array covering the Frontal Cortex. **(A)** 2D Schematic of Channel Layout (15 optode emitters and 16 optode detectors forming 48 channels). **(B)** 3D Schematic of Channel Layout (Pink represents optode emitters, blue represents optode detectors).

The 48-channel system employed in this study consisted of 15 optode emitters and 16 optode detectors, with an average distance of 3 cm (range: 2.7–3.3 cm) between each emitter and detector pair, covering the frontal cortex. Each connection between an adjacent NIR source and detector was defined as a “channel,” with channel locations determined according to the Brodmann partition, as illustrated in Figure 1. Previous studies have identified working memory and decision-making as fundamental components of cognition, with the prefrontal cortex playing a pivotal role in these processes (Murray et al., 2017). Notably, the dorsolateral prefrontal cortex exhibits heightened activation during information integration and processing (Prabhakaran et al., 2000). Consequently, the prefrontal cortex was selected as the primary region of interest in this study. The investigated areas also encompassed the temporal, parietal, frontal, and frontal eye fields, which are intricately associated with voluntary behavior and the management of open-ended situations. These regions are particularly critical to monitor in naturalistic environments where complex cognitive processes dynamically unfold (Stuss and Knight, 2013).

### 2.3 Experimental design and material

#### 2.3.1 Experimental design

The experiment employed a one-factor, two-level between-subjects design, in which the independent variable was the format of learning material presentation, categorized into two conditions: spatial proximity and spatial separation. The behavioral dependent variables included retention scores, transfer scores, total scores, and cognitive load, with mental effort serving as the primary indicator and study time as a secondary measure. The neurological dependent variable was the relative change in oxygenated hemoglobin (HbO) levels during the learning phase.

#### 2.3.2 Experimental material

**Figure 2:**
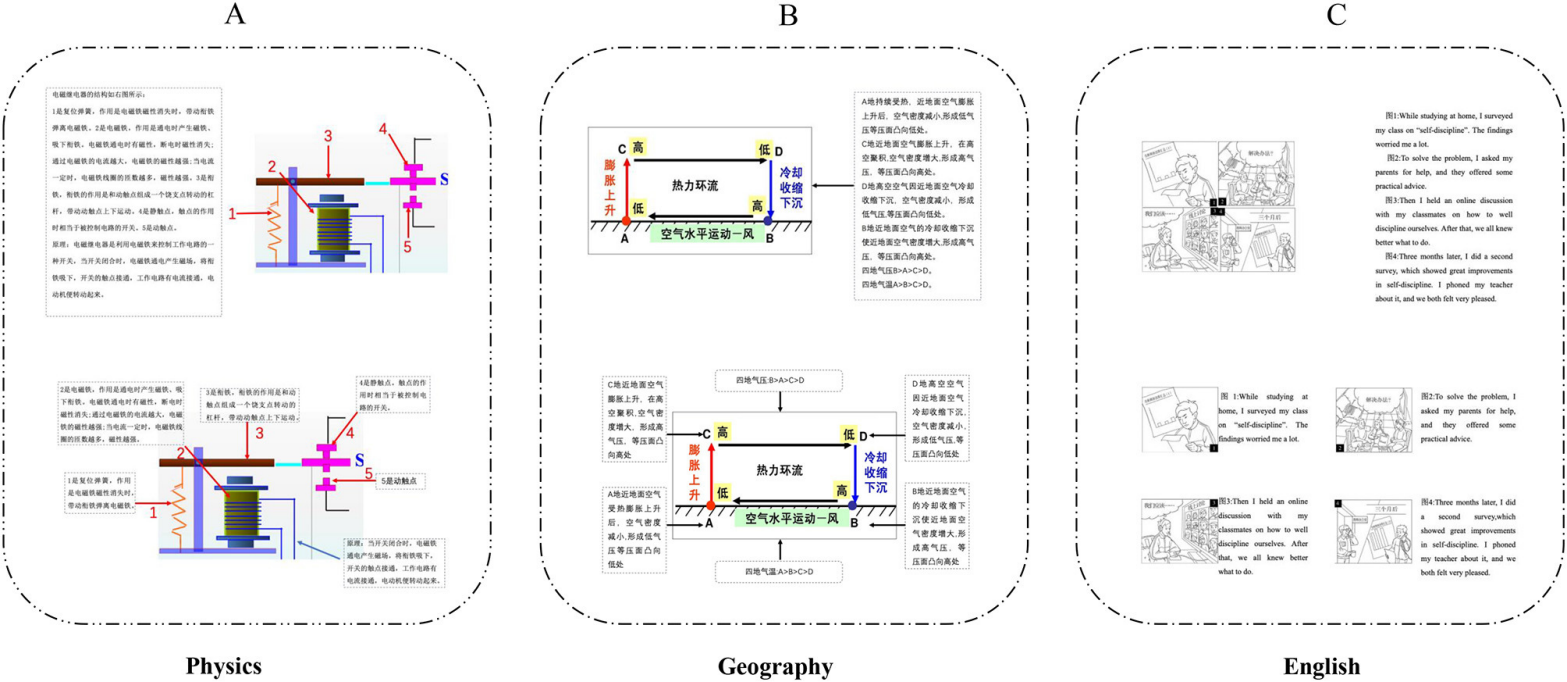
Examples of learning materials for various disciplines. The upper half shows a diagram of spatial proximity groups, and the lower half shows a diagram of spatial separation groups. **(A)** Physics. **(B)** Geography. **(C)** English.

The learning materials consisted of text and images covering three subjects: English, Physics, and Geography. In educational practice, multimedia teaching materials are widely utilized in English, Geography, and Physics, typically integrating text and images. Reading comprehension and writing in English frequently involve the integration of diagrams and text; spatial analysis and map interpretation in Geography also necessitate the integration of graphics and text; similarly, experiments and theoretical explanations in Physics typically require graphic-text integration. Given the high demand for graphic-text integration in educational design across these three disciplines, they were selected as the focus of this study. As illustrated in Figure 2, the experimental materials (Physics, Geography, and English) were presented in two formats: adjacent and separated, organized into five blocks. The English materials were selected from the College Entrance Examination English (Look and Talk type). The Physics materials focused on the composition and principles of electromagnetic relays, while the Geography materials addressed the principles of thermal circulation. Sweller's explanation of the level of elemental interactivity refers to the degree of element-tallness at which a task can be learned meaningfully without having to learn the relationship between any other elements (Sweller, 1994). Therefore, Physics is categorized as high elemental interactivity material, whereas Geography and English are considered low elemental interactivity materials. The selected English and Geography graphic-text combinations exhibit high redundancy and low elemental interactivity, whereas the Physics graphic-text combinations display low redundancy and high elemental interactivity. To investigate the influence of spatial proximity effects, the pictures and texts in the learning materials were adjusted in distance between the two groups, with all other factors held constant to control for potential confounding variables. The quiz comprised multiple-choice questions, including four retention questions and one to two transfer questions, each worth one point, totaling 26 points. A pretest was administered prior to the experiment to ensure that participants' familiarity with the experimental materials was sufficiently low, thereby minimizing the influence of prior knowledge as a confounding factor.

Retention scores assessed learners' ability to remember and comprehend the content, reflecting the accuracy of their knowledge retention. The test included twelve retention questions, such as: “What can change the strength of an electromagnet's magnetism?” The transfer test evaluated learners' ability to apply and generalize knowledge and consisted of four questions. An example question was: “The following poem describes a phenomenon that the principle of thermal circulation can explain.”

Cognitive load was measured using the Mental Task Load Scale (MTLS), which consisted of two primary components: an evaluation of material difficulty and an assessment of required mental effort. A 9-point Likert scale was used, ranging from 1 (“very easy”) to 9 (“very difficult”). Higher scores indicated greater cognitive load. Participants were instructed to select a number between 1 and 9 that best reflected their perceived cognitive load.

### 2.4 Experimental procedure

**Figure 3:**
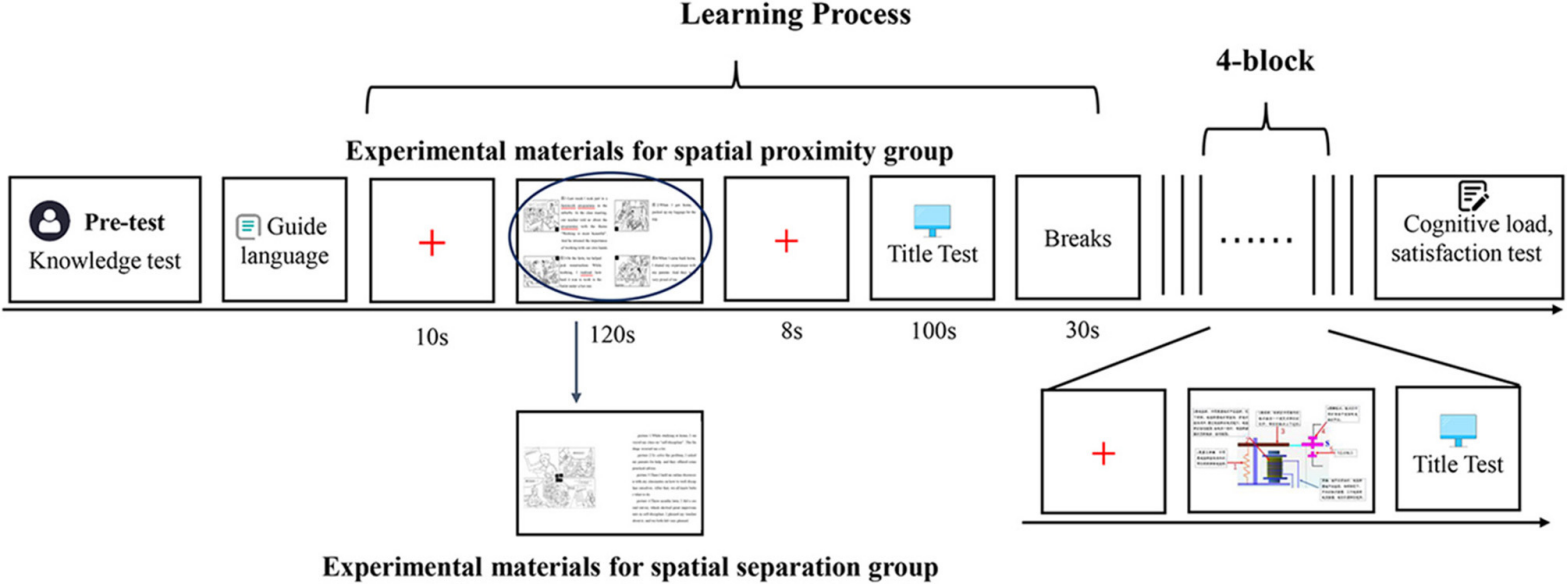
Experimental flowchart. “+” represents the “resting gaze point” stage (the purpose of which is to return the blood oxygen level in the subject's brain to baseline). “...” represents “repeated learning and testing sessions.” 4-block represents “the experiment is conducted four times.”

The experimental program was developed using E-Prime 3.0 software, with stimuli presented on a 23-inch monitor at a resolution of 1,280 × 768 pixels. The experiment consisted of five blocks, with a total duration of approximately 25 min. Initially, participants received instructions describing the experimental procedure. Subsequently, participants were randomly assigned to one of two experimental conditions: spatial proximity or spatial separation. The learning materials were categorized into three subjects: english, Physics, and Geography, with a learning duration of 2 min for each material within a block. At the end of each learning block, a 10-s red fixation cross (“+”) was displayed at the center of the screen to allow the participants' cerebral blood oxygen levels to return to baseline. Following the resting period, participants completed three to four retention questions and one to two transfer questions to assess their behavioral responses. Answers were entered using an alphabetic keyboard (e.g., pressing the “A” key for answer “A,” the “B” key for answer “B,” etc.). Participants were instructed to use their right hand for all response operations on a standard keyboard to ensure consistent right-hand motor preparation across all experimental conditions. Completion of the answers marked the end of each block, with each block lasting approximately three and a half minutes. Before proceeding to the next block, participants underwent a 30-s resting period to promote relaxation. The appearance of the prompt “About to enter the next learning content” on the screen signaled the beginning of a new experimental block. Upon completion of the experiment, participants completed the cognitive load and satisfaction measurement scales. The detailed procedure is illustrated in Figure 3.

### 2.5 Data analysis procedure

#### 2.5.1 fNIRS data analysis

The NIR data were pre-processed using NIRSpark software. The experimental pre-processing steps were as follows: ①Irrelevant periods prior to the official start of the experiment were removed, and a preliminary inspection of all 48 channels in each dataset was conducted to eliminate poor-quality data. ②Motion artifacts were corrected using a spline interpolation algorithm on a channel-by-channel basis. This interpolation method is commonly used for motion artifact correction. The algorithm identified potential artifacts based on input parameters and automatically extracted intervals where artifacts were likely to occur, with the advantage of localizing and correcting only the affected segments. ③The data were band-pass filtered at 0.01–0.2 Hz to remove physiological noise, including head movements, heartbeat, respiration, and low-frequency signal drift. ④Optical density data were converted to hemoglobin concentration values using the modified Beer–Lambert law. ⑤The hemoglobin concentration data obtained after preprocessing were averaged by superposition to yield group block averages. The hemodynamic response function was derived by superimposing and averaging the block paradigms to reduce random noise associated with the task. ⑥The general linear model (GLM) was applied to analyze the HbO time-series data. By fitting the experimental data to the experimental design, the GLM yielded activation coefficients (β values), reflecting the degree of activation in each channel (Plichta et al., 2007). HbO has been demonstrated to exhibit a higher signal-to-noise ratio, making it more sensitive to cerebral blood flow changes. Therefore, all subsequent analyses and evaluations were conducted using HbO data (Ma et al., 2016). For HbO signals, positive β-values indicate an increase in task-related cortical activation, whereas negative β-values indicate a decrease. β-values were used as indicators of activation in the corresponding brain regions. Subsequently, single-sample *t*-tests and analyses of variance were performed on the β-values under different conditions. The statistical results were corrected for multiple comparisons using the false discovery rate (FDR) method. Corrected *p*-values < 0.05 were considered statistically significant, thereby reducing the false positive rate (Noble, 2009).

#### 2.5.2 Statistical analyses

Behavioral and preprocessed NIR data were statistically analyzed using SPSS 25.0, with the significance level set at 0.05. Descriptive statistics were reported as mean ± standard deviation. Two-sample independent *t*-tests were performed to compare subjects' reaction times, retention scores, and other variables across different graphic presentation formats. Bootstrap tests were also conducted. Initially, the NIR data were analyzed using one-sample *t*-tests and independent two-sample *t*-tests for the β-values under the spatial proximity and spatial separation conditions. Additionally, Bootstrap tests were conducted to identify significant (*p* < 0.05) passages for further analysis. The statistical results were corrected for multiple comparisons between channels using the false discovery rate (FDR) method. Subsequently, the impact of different disciplines on the multimedia spatial proximity effect was examined using a 2 (graphic distance: separation, proximity) × 3 (discipline: geography, physics, English) repeated-measures ANOVA on the β-values for each channel in the prefrontal cortex. Bonferroni corrections were applied for significant channels (*p* < 0.05), followed by further simple effects analyses of channels exhibiting significant interaction effects.

## 3 Research result

### 3.1 Behavioral experiment results

After conducting preprocessing checks on signal quality, one participant was excluded due to excessive artifacts caused by large head movement amplitudes, while another participant was excluded due to signal quality interruptions during the experiment. Ultimately, 34 datasets were included in the analysis, with 17 participants in each of the spatial proximity and spatial separation groups.

Two-sample independent *t-*tests were conducted to analyze reaction time (RT), retention performance, migration performance, cognitive load, and accuracy rate (ACC) across different graphic presentation formats. Additionally, bootstrap tests were applied, and the results of the analyses are presented in Table 2. The spatial proximity and spatial separation groups showed differences in performance maintenance {*t* (34) = 1.95, *p* = 0.57, 95% CI [−1.64, −0.44]}, accuracy {t (34) = 3.05, *p* = 0.005, 95% CI [−0.16, −0.37]}, and reaction time {*t* (34) = 2.22, *p* = 0.034, 95% CI [0.12, 1.47]) (*p* < 0.05)}. No statistically significant differences were found in transfer performance {*t*(34) = 3.3, *p* = 0.002, 95% CI [−2.827, −0.79]} and cognitive load {*t*(34) = 0.16, *p* = 0.546, 95% CI [−0.94, 1.8]) (*p* > 0.05)}.

**Table 2:** Descriptive statistics table, *t*-test, and Bootstrap results of the results under different material presentations.

**Variable**	**Group**	**M ±SD**	***t***	***P***	***SE***	***P***	**Lower 95% CI**	**Upper 95%CI**
Response time	Separate groups	7.99 ± 0.95	2.22	0.03	0.03	0.03	0.12	1.47
	Proximity group	8.75 ± 1.04						
Maintaining performance	Separate groups	2.65 ± 0.99	3.3	0.00	0.30	0.00	−1.64	−0.44
	Proximity group	1.59 ± 0.84						
Migration results	Separate groups	14.71 ± 2.26	1.95	0.06	0.70	0.06	−2.83	−0.79
	Proximity group	16.12 ± 1.97						
Cognitive load	Separate groups	13.41 ± 1.5	0.61	0.55	0.69	0.55	−0.94	1.80
	Proximity group	13.00 ± 2.3						
Correct rate	Separate groups	0.62 ± 0.11	3.05	0.00	0.33	0.01	−0.16	−0.37
	Proximity group	0.72 ± 0.08						

A 2 (graphic distance: separation, proximity) × 3 (discipline: geography, physics, English) repeated measures ANOVA was conducted to analyze the participants‘ retention and transfer scores (For English language participants, the data used the average correct rate for the three blocks). The results revealed a significant main effect of discipline on retention scores (*F* = 49.74, *p* < 0.000, partial η^2^ = 0.76), but no significant interaction was found between subject and graphic distance. Bonferroni-corrected *post hoc* tests further revealed that the retention scores of the proximity groups across all disciplines (English, Physics, and Geography) were significantly higher than those of the separation groups (Physics proximity: 0.69, Physics separation: 0.55; Geography proximity: 0.48, Geography separation: 0.38; English proximity: 0.87, English separation: 0.83). These results suggest that, across disciplines, the proximity condition enhances learners' retention scores more effectively than the separation condition.

A significant main effect of subject was observed for transfer scores (*F* = 24.64, *p* < 0.000, partial η^2^ = 0.61). Within the proximity group, a significant difference was observed between English and Physics (*p* < 0.000), with Physics yielding higher transfer scores than English (0.9 ± 0.05 vs. 0.4 ± 0.04). Additionally, a significant difference was found between Physics and Geography (*p* = 0.003), with Physics outperforming Geography (0.9 ± 0.05 vs. 0.5 ± 0.08). No significant difference was found between Geography and English (0.5 ± 0.08 vs. 0.4 ± 0.04). These findings suggest that Physics, characterized by higher material interactivity, benefits more from the proximity condition, thereby facilitating better transfer learning compared to Geography and English, which involve lower material interactivity.

### 3.2 Brain imaging results

#### 3.2.1 Analysis of multimedia spatial proximity effect

**Figure 4:**
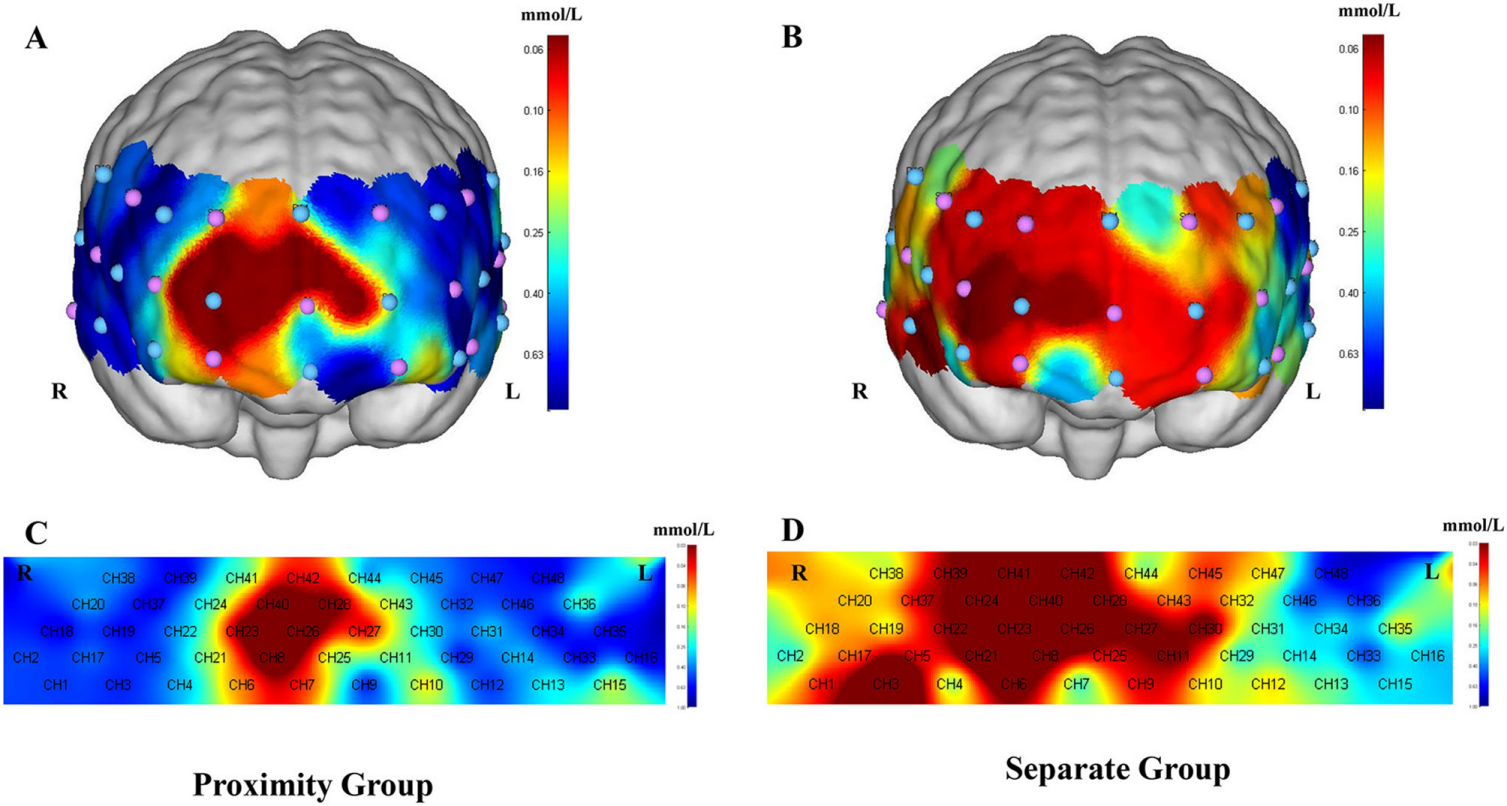
Significant activation of HbO in different brain regions under different conditions. **(A)** Significant activation channels in the spatial proximity group (3D image), primarily in the frontal lobe. **(B)** Significant activation channels in the spatial separation group (3D image), primarily in the temporal lobe and frontal lobe. **(C)** Significant activation channels in the spatial proximity group (2D image). **(D)** Significant activation channels in the spatial separation group (2D image).

A one-sample *t*-test was conducted on the β-values of each channel at different graphic distances, using a test value of 0. The results revealed that, under the spatial separation condition, the channels that were significantly activated included CH1, CH3, CH4, CH6, CH7, CH8, CH9, CH11, CH21, CH22, CH23, CH24, CH26, CH27, CH28, CH29, CH30, CH35, CH37, CH39, CH40, CH41, and CH42 (*p* < 0.05). The activated brain regions were primarily located in the temporal and frontal lobes, with the temporal lobe involved in processing auditory information, language comprehension, and memory functions. The frontal lobe, situated in the anterior part of the brain, is responsible for executive functions, problem-solving, and planning. Under the spatial proximity condition, significant activation was observed in channels CH7, CH8, CH23, CH26, CH27, CH28, CH40, and CH42. These activated regions were primarily in the frontal lobe, while the remaining channels showed no significant activation (*p* > 0.05), as illustrated in Figure 4.

**Figure 5:**
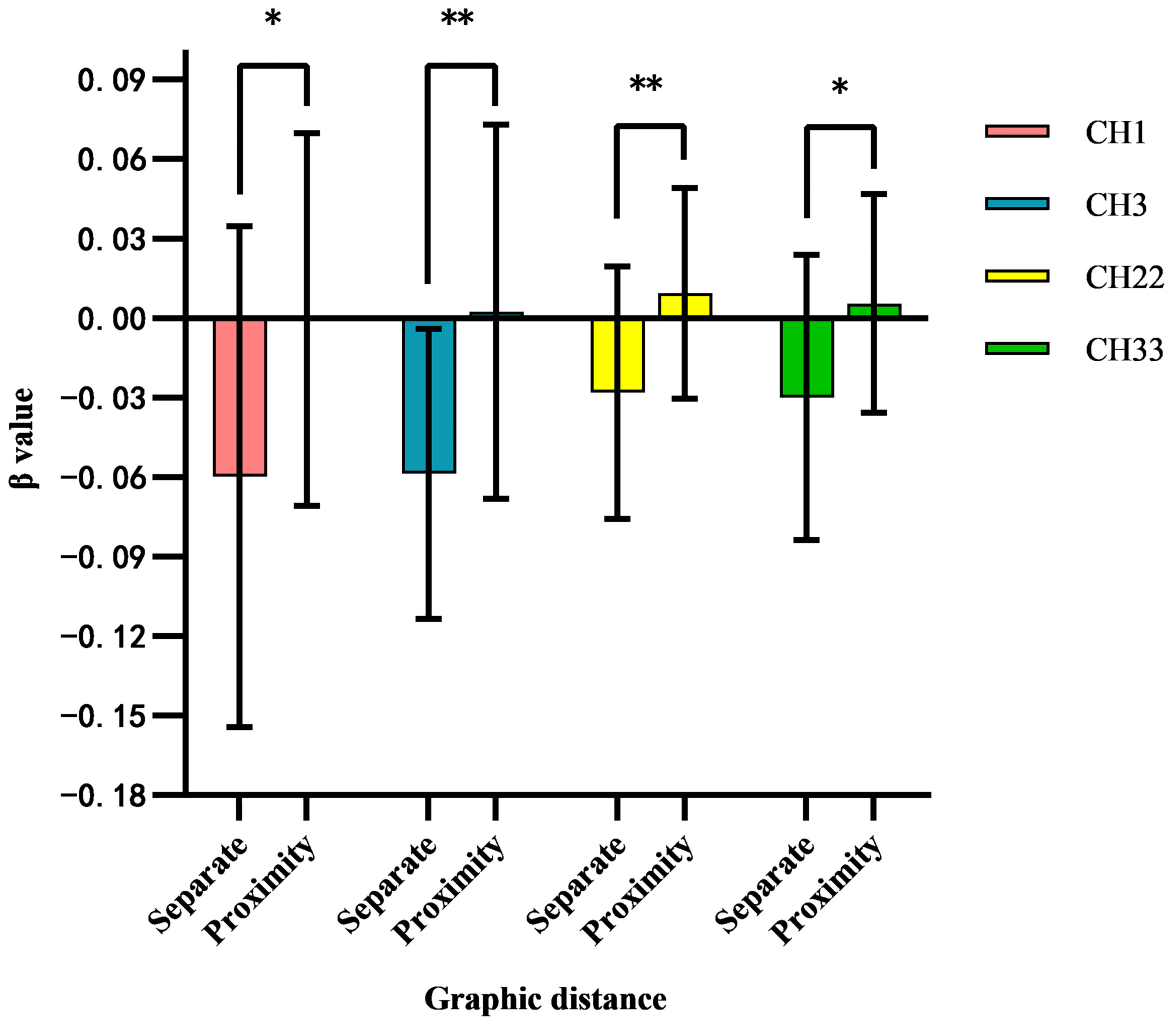
Differences in HbO activation in channel 1 (middle temporal gyrus), channel 3 (frontal pole), channel 22 (dorsolateral prefrontal cortex), and channel 33 (middle temporal gyrus) under spatial separation and spatial proximity conditions. ^*^p < 0.05, ^**^p < 0.01.

Independent two-sample *t*-tests were performed to analyze the β-values of each channel under the two conditions, with the results presented in Table 3 and Figure 5 (only channels exhibiting significant differences are shown). The findings indicated significant differences in the β-values of channels CH1, CH3, CH22, and CH33 under the spatial proximity effect condition (separation × proximity). These differences corresponded to brain regions such as the middle temporal gyrus, frontal pole region, and dorsolateral prefrontal cortex. Notably, activation levels in the cortices associated with these channels were significantly higher in the proximity group compared to the separation group, suggesting that the middle temporal gyrus, frontal pole region, and dorsolateral prefrontal cortex exhibit greater activation under the proximity condition than under the separation condition.

**Table 3:** Differential brain area channeling in separate group and proximity group.

**Channel**	**Brain regions**	***T***	***p***	**M**	**SD**
CH1	Middle temporal gyrus	−2.074	0.04	−0.001	0.095
CH3	Frontopolar area	−2.821	0.00	0.002	0.071
CH22	Dorsolateral prefrontal cortex	−2.491	0.01	0.009	0.039
CH33	Middle temporal gyrus	−2.162	0.03	0.006	0.041

#### 3.2.2 The spatial proximity effect between different disciplines

**Figure 6:**
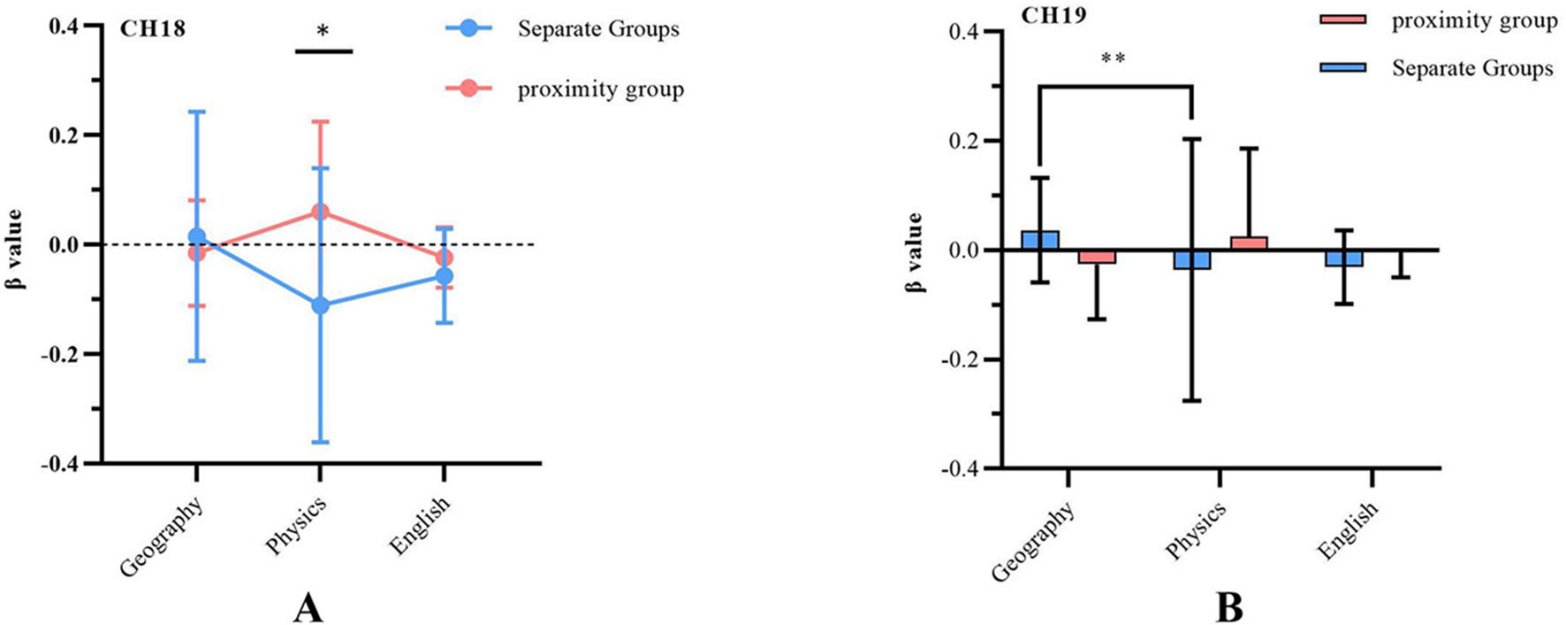
Significant interaction between subject and spatial proximity effect in brain region channels. **(A)** Differences in HbO in CH18 (superior temporal gyrus) under spatial separation and proximity conditions. **(B)** Differences in HbO in CH19 (Broca's area) under spatial separation and proximity conditions. *p < 0.05, **p < 0.01.

A 2 (graphic distance: separation, proximity) × 3 (discipline: geography, physics, English) repeated-measures ANOVA was conducted on the β-values of the 48 channels. A significant interaction effect between discipline type and graphic distance was observed on channels CH17, CH18, CH19, and CH46 (CH17: *F* = 4.03, *p* = 0.02, η^2^ = 0.207; CH18: *F* = 3.58, p = 0.04, η^2^ = 0.101; CH19: *F* = 5.34, *p* = 0.01, η^2^ = 0.256; CH46: *F* = 3.45, *p* = 0.03, η^2^ = 0.097). These results indicated differences in brain activation levels between subjects in the separation and proximity groups when learning geography, physics, and English, suggesting that the proximity effect on brain activation varies across disciplines. Further analysis of the channels exhibiting significant interaction effects was conducted through simple effects analysis, as shown in Figure 6. A significant difference in the change of HbO concentration between the proximity and separation conditions was observed at CH18 (superior temporal gyrus) during physics learning, across the three disciplinary conditions (*F* = 5.528, *p* = 0.02, η^2^ = 0.147). Specifically, the proximity condition (0.6 ± 0.16) exhibited significantly higher HbO concentrations than the separation condition (−0.11 ± 0.25). These results suggest that the superior temporal gyrus (CH18) is particularly sensitive to the processing mechanisms associated with the spatial proximity effect. In contrast, no significant differences were observed in the changes of HbO concentration between the proximity and separation conditions in either the English (*F* = 1.82, *p* = 0.18, η^2^ = 0.05) or geography (*F* = 0.26, *p* = 0.61, η^2^ = 0.008) disciplines.

Moreover, no differences were observed in the effects of physics, geography, and English on brain activation under the proximity condition. However, a significant difference in the change of HbO concentration in Broca's area (CH19) was observed between physics and geography under the separation condition (*F* = 6.17, *p* = 0.004, η^2^ = 0.28). Activation was significantly higher when studying geography (0.03 ± 0.2) compared to studying English (−0.3 ± 0.1), with no significant differences between the two conditions for the other disciplines.

## 4 Discussion

This study utilized functional near-infrared spectroscopy (fNIRS) to examine the effects of spatial proximity on multimedia learning outcomes and associated neural mechanisms. The influence of discipline type on the spatial proximity effect was explored to elucidate underlying neural processes. Behaviorally, learners in the spatial proximity condition achieved significantly higher accuracy than those in the spatial separation condition. Across all disciplines, the proximity group demonstrated superior retention test performance. Neurally, spatial proximity evoked greater activation in the middle temporal gyrus (MTG), frontal pole (FPC), and dorsolateral prefrontal cortex (dlPFC) compared to spatial separation. Furthermore, discipline type significantly modulated neural responses to spatial proximity. For high-interactivity disciplines (e.g., physics), the proximity condition demonstrated significantly greater superior temporal gyrus (STG) activation than the separation condition. In low-interactivity disciplines (e.g., geography and English), however, no significant activation differences emerged in corresponding brain regions.

### 4.1 Spatial proximity effects on learning outcomes

This study found that learners in the spatial proximity group performed significantly better than those in the spatial separation group (ACC_separation < ACC_proximity, RT_separation < RT_proximity), indicating that spatial proximity can effectively enhance learning outcomes and reduce the cognitive burden associated with visual search and information integration (de Koning et al., 2020). These results support Hypothesis H1 and align with Mayer's findings. Mayer (2009) emphasized that when text and images are presented separately in space, learners must expend considerable cognitive resources to search for relevant information, which interferes with the integration and processing of text and images, ultimately diminishing learning effectiveness (Mayer, 2009). Presenting text and images nearby, both visually and temporally, is therefore more conducive to efficient text-image processing, promoting knowledge comprehension and memory retention. These findings are also consistent with the empirical results of Florax and Rolf (2010), which demonstrated that the spatial separation of text and images requires learners to frequently shift attention between disparate information sources, thereby increasing extraneous cognitive load, constraining working memory capacity, and ultimately impairing learning effectiveness (Florax and Rolf, 2010).

Although the differences in transfer performance and subjective cognitive load between the groups did not reach statistical significance, the separation group exhibited higher scores than the proximity group on both measures. This result aligns with the findings of Cammeraat et al. (2020) regarding the spatial proximity effect, suggesting that while spatial proximity may influence cognitive load, it does not easily produce statistically significant differences. This may be attributed to the observation that the spatial proximity effect tends to be more pronounced when material complexity is high, redundancy is low, and learners' working memory capacity is limited (Cierniak et al., 2009). Although the proximity of text and images helps reduce cognitive load, its effectiveness may be limited when the learning materials are not highly interactive. Furthermore, cognitive load during learning can be categorized into intrinsic load (material complexity), extraneous load (instructional design flaws), and germane load (schema construction), with extraneous and germane loads being particularly influenced by instructional design (Hagoort et al., 2009; Klepsch and Seufert, 2020). The present study utilized only a single subjective scale to measure overall cognitive load without distinguishing among its different types, which may have reduced measurement sensitivity and impacted the ability to detect significant differences between groups. Future research should further differentiate the types of cognitive load, employ multidimensional measurement approaches, and integrate the spatial proximity effect to verify their causal relationship.

### 4.2 The effect of spatial proximity effect on learners' brain activation level

According to the HbO results under spatial separation and spatial proximity conditions, the brain regions significantly activated under spatial separation included the temporal lobe and frontal lobe, specifically the middle temporal gyrus, temporal pole, inferior frontal gyrus, orbitofrontal cortex, and dorsolateral prefrontal cortex. Under spatial proximity conditions, the significantly activated regions were primarily concentrated in the frontal lobe, including the orbitofrontal cortex, frontal pole, and dorsolateral prefrontal cortex. These findings support Hypothesis H2, suggesting that spatial separation conditions significantly increase activation in brain regions associated with executive control and working memory. Previous studies have demonstrated that activation in the dorsolateral prefrontal cortex and frontal pole is more pronounced during tasks involving information retention and integration (Xiao et al., 2020). Additionally, the temporal lobe plays a crucial role in integrating information from different sensory channels (Barker, 2024), performing in-depth semantic analysis, and separately encoding text and images. The broader activation of the temporal lobe and dorsolateral prefrontal cortex under spatial separation conditions further suggests that the spatial separation of text and images increases cognitive load during the integration process. Due to the spatial separation of text and image information, learners must continuously maintain memory representations while frequently shifting attention between different information sources. This process increases the load on executive control and working memory, resulting in broader neural activation.

Additionally, under the text-image spatial proximity condition, activation intensity in the middle temporal gyrus, dorsolateral prefrontal cortex, and frontal pole was significantly higher compared to the separation condition, further supporting Hypothesis H3. The dorsolateral prefrontal cortex is primarily responsible for maintaining and processing visual-spatial working memory (Courtney et al., 1998; Pinti et al., 2020), while the frontal pole optimizes the encoding and integration of multimodal information through functions such as goal management, resource allocation, and conflict monitoring (Rolls et al., 2024). Semantic encoding and retrieval primarily rely on the left prefrontal and temporal lobes (Wang et al., 2009; Xiao et al., 2020), with the middle temporal gyrus playing a central role in semantic retrieval and processing (Davey et al., 2016). These brain regions are closely associated with semantic integration (Wang et al., 2024), multimodal information integration, and working memory (Segal and Elkana, 2023). Therefore, the enhanced activation of brain regions in the spatial proximity condition may result from the reduced need for additional visual scanning and searching, allowing immediate matching of graphic and textual information during the visual input stage. This facilitates the simultaneous retention of more information in working memory, improves the efficiency of semantic integration, and promotes deep knowledge construction. This finding aligns with the multimedia learning cognitive theory, which posits that “the more integration processing, the better the outcomes,” providing robust support for the neural basis of the spatial proximity effect. In summary, instructional design should adhere to cognitive processing principles by arranging text and image information in a coherent spatial layout, promoting multi-sensory collaborative processing, and optimizing working memory resource allocation to enhance the efficiency of complex information processing and facilitate deep learning. Future development of teaching resources should fully consider information integration pathways and prioritize multimedia presentation designs that minimize cognitive load, thereby supporting learners in constructing adequate and coherent mental representations.

### 4.3 The influence of discipline on the spatial proximity effect

The spatial proximity effect has been demonstrated to be effective across several disciplinary domains (de Koning et al., 2020; Makransky et al., 2019). However, most studies have relied on subjective measurements and lacked neural-level data to substantiate their findings. Based on behavioral indicators and fNIRS data, this study investigated the neural processing mechanisms underlying the spatial proximity effect across three major disciplines: english, physics, and geography. The results indicated that the proximity groups in all three disciplines demonstrated significantly higher accuracy rates and faster reaction times, thereby supporting hypothesis H4 and aligning with previous research demonstrating that spatial proximity enhances information integration efficiency (Castro-Alonso et al., 2021; Vu et al., 2021). Further analysis revealed a significant interaction between subject type and the spatial proximity effect in Broca's area and the superior temporal gyrus (STG), consistent with previous research on the functional localization of language processing and semantic integration regions (Bulut, 2023). In physics, HbO activation in the superior temporal gyrus was significantly greater under spatial proximity conditions compared to separation conditions, with learners' accuracy rates improving concurrently. This finding supports the theoretical hypothesis that highly interactive materials facilitate semantic integration. In contrast, although the proximity groups in English and geography exhibited superior learning outcomes, activation differences in the relevant brain regions did not reach statistical significance. This discrepancy may be attributed to the lower interactivity of the materials in these subjects, which emphasize memory retrieval and reproduction processes. The present results provide neuroscientific evidence supporting the spatial proximity effect in highly interactive disciplines (such as physics), further validating that highly interactive materials are more likely to induce significant spatial proximity benefits (Wang et al., 2016), thereby promoting the integration of text and image information. Based on these findings, instructional design should carefully consider differences in material interactivity, appropriately apply the spatial proximity principle, and optimize multimodal resource allocation to reduce extraneous cognitive load and enhance learning efficiency. For different material properties, an “interactivity-proximity matching” strategy can be adopted: highly interactive materials should employ a design approach emphasizing the proximity of text and images, whereas for less interactive materials, spatial proximity requirements may be moderately relaxed. Future research may be expanded to include additional disciplines and task types, systematically exploring the generalizability of the spatial proximity effect across diverse learning contexts and providing more comprehensive neuroscientific evidence supporting multimedia learning cognitive theory.

### 4.4 Limitations and future directions

This study provides empirical support for the spatial proximity effect in multimedia learning cognitive theory from a neuroscientific perspective. However, the results require further verification through additional independent samples and diverse experimental paradigms to ensure the stability and generalizability of the conclusions. It is important to note that, due to the limitations in spatial resolution and detection depth of fNIRS technology, this study was able to capture only blood oxygenation changes in the superficial cortical areas associated with the task. Future studies should employ multimodal brain imaging techniques (such as EEG, fMRI, and eye tracking) in combination to further enrich the evidence regarding the neural mechanisms of the spatial proximity effect. This approach also provides a crucial means to replicate and validate the current findings across different technical modalities.

Additionally, the sample in this study consisted of college students with low levels of prior knowledge, resulting in a relatively homogeneous sample structure. Future research should expand the sample size to include learners with varying knowledge backgrounds, learning styles, and age groups, particularly primary and secondary school students as well as elderly learners, to more comprehensively assess the applicability and educational value of the spatial proximity effect across diverse learning populations. Although this study controlled for the overall knowledge level of learners through pre-testing, micro-level differences in knowledge structure were not analyzed in depth. Future studies should employ more sophisticated tools for measuring knowledge construction to explore the interactive relationship between individual knowledge structures and the spatial proximity effect, thereby deepening the understanding of individual differences in multimedia learning mechanisms.

In summary, future research should continue to validate and expand the applicability of the spatial proximity effect from the perspectives of technology, sampling, and variable control, thereby providing stronger empirical support for personalized and diversified instructional design.

## 5 Conclusions

Based on behavioral and brain imaging results, this study systematically investigated the impact of the spatial proximity effect on college students' learning cognition and its underlying neural mechanisms. The results indicate that learners in the spatial proximity group achieved significantly higher accuracy rates than those in the spatial separation group, further confirming that the proximity of text and images can effectively enhance learning outcomes. Additionally, brain activation in the middle temporal gyrus, dorsolateral prefrontal cortex, and frontal pole was significantly higher under spatial proximity conditions, revealing the neural basis of the spatial proximity effect on learning cognition. Further analysis across different disciplines revealed that spatial proximity positively influenced both retention and transfer learning in the three core subjects: physics, geography, and English. In highly interactive disciplines, such as physics, the facilitative effect of spatial proximity on transfer learning was more pronounced, accompanied by significantly increased activation in the superior temporal gyrus. This finding indicates that the interactivity of disciplinary materials plays a critical regulatory role in the spatial proximity effect. In summary, this study verified the spatial proximity effect in college students' learning from a neuroscientific perspective and further elucidated the regulatory role of disciplinary material interactivity in the spatial proximity effect. The findings not only expand the cognitive neuroscientific understanding of the spatial proximity effect but also provide empirical support for instructional design in multimedia learning. Instructional designers should fully consider individual differences in learners' working memory capacity, optimize the presentation of text and images, and effectively allocate multimodal learning resources to reduce extraneous cognitive load and enhance learning efficiency. Future research should further refine the interactivity levels of subject materials, expand sample diversity, integrate multiple neuroimaging techniques, and explore the applicability of the spatial proximity effect across a broader range of learning contexts, including its long-term effects.

## Data Availability

The raw data supporting the conclusions of this article will be made available by the authors, without undue reservation.
